# Genetic diversity and population structure of Mexican Gir cattle using genome-wide SNP data

**DOI:** 10.1093/tas/txag094

**Published:** 2026-07-17

**Authors:** Ricardo Martínez-Rocha, Jonathan Emanuel Valerio-Hernández, Rafael Núñez-Domínguez, Rodolfo Ramírez-Valverde, Joel Domínguez-Viveros, Juan Fernando Saiz, Julius Mugambe, Jorge Hidalgo

**Affiliations:** Posgrado en Producción Animal, Universidad Autónoma Chapingo, Texcoco, State of Mexico 56230, Mexico; Facultad de Medicina Veterinaria y Zootecnia, Universidad Nacional Autónoma de Méxio, Ciudad Universitaria, Mexico City, 04510, Mexico; Posgrado en Producción Animal, Universidad Autónoma Chapingo, Texcoco, State of Mexico 56230, Mexico; Posgrado en Producción Animal, Universidad Autónoma Chapingo, Texcoco, State of Mexico 56230, Mexico; Facultad de Zootecnia y Ecología, Universidad Autónoma de Chihuahua, Chihuahua, 31000, Mexico; Facultad de Zootecnia y Ecología, Universidad Autónoma de Chihuahua, Chihuahua, 31000, Mexico; Asociación Mexicana de Criadores de Cebú, Tampico, 89100, Mexico; Department of Animal Biosciences, Interbull Centre, Uppsala, 75007, Sweden; Department of Animal and Dairy Science, University of Georgia, Athens, GA 30602, United States

**Keywords:** *Bos indicus*, genomic inbreeding, runs of homozygosity

## Abstract

This study characterizes the genomic diversity and population structure of Mexican Gir cattle. We sampled 226 animals from three tropical herds and isolated DNA from hair samples was genotyped using the GGP Indicus 50K array. After quality control, 212 animals and 28,394 autosomal SNPs remained for a within-breed analysis. We merged the Mexican Gir dataset with an available online database involving 134 worldwide breeds. The combined dataset was used for across-breed comparisons, with 9091 shared SNPs. Within-breed diversity was high (Heterozygosity Observed = 0.40; Heterozygosity Expected = 0.38), and genomic inbreeding by Hardy-Weinberg Equilibrium ($F_{IS}=-0.04$), by Genomic Relationship Matrix ($F_{\mathrm{GRM}}=-0.02$) and by Runs of Homozygosity ($F_{\mathrm{ROH}}=0.07$) was low. Runs of Homozygosity were dominated by short segments (1–2 Mb), which accounted for 75% of the total detected segments, consistent with historical rather than recent inbreeding. Multidimensional scaling grouped Mexican Gir with Asian zebu, particularly Gir from India and Pakistan; pairwise Fixation Index values were lowest versus Gir, Red Sindhi, Lohani, Rojhan, and Tharparkar, and IBS heatmaps showed greater similarity to Asian indicine than to unrelated zebu. ADMIXTURE-supported ancestry components were concordant with the breed’s introduction history. Overall, Mexican Gir exhibit ample genetic variability and low recent inbreeding, providing a robust foundation to intensify selection for productivity and tropical adaptation while safeguarding diversity. These results support genomic monitoring ($F_{IS}$, $F_{\mathrm{GRM}}$, $F_{\mathrm{ROH}}$), balanced sire usage, and targeted germplasm introduction as management priorities.

## Teaser

Mexican Gir cattle retain high genetic diversity and low recent inbreeding while preserving strong genetic ties to their South Asian origins. These results support the sustainable use of genomic tools to improve productivity without compromising their long-term genetic diversity.

## Introduction

The Gir cattle breed (*Bos indicus*) originated in the Rajputana and Baroda regions of India, particularly in the Gir Hills, where it developed under high temperatures and nutritional constraints (AMCC 2021). The animals were selected artificially for productivity under those selective pressures, yielding a breed with remarkable heat tolerance, productive efficiency, and dual-purpose capabilities, characteristics that propelled its spread beyond the Indian subcontinent to other tropical regions of the world (Filho et al. 2015).

During the 20th century, Gir cattle were introduced and widely distributed in Brazil, where they became one of the main Zebu breeds and underwent intensive selection via genetic improvement programs, particularly focused on milk production and performance in tropical environments (Filho et al. 2015). As a result of this process, the Brazilian Gir became a key genetic source for other Latin American countries. In Mexico, the Gir population was established from animals imported from Brazil starting in the 1940s, with subsequent additions of germplasm of U.S. and Brazilian origin in different decades, to broaden the genetic base and strengthen the breed’s productive performance under tropical conditions (CCG 2025).

Currently, the Gir breed is of increasing importance in the Mexican tropics, particularly in systems focused on growth and beef production. In this context, the Mexican Association of Zebu Breeders (AMCC 2021), in collaboration with the Autonomous University of Chihuahua, has implemented periodic genetic evaluations and programs such as the Weight Development Control Program (PCDP), which focuses on identifying animals with the greatest genetic potential for weight gain (Martínez González et al. 2021; Domínguez Viveros 2025). However, as is the case in many livestock populations subjected to directional selection, the intensification of breeding programs can lead to a progressive reduction in genetic variability if not properly monitored and managed.

Genetic diversity and population structure are fundamental components for the sustainability of breeding programs, as they determine the potential response to selection, adaptability to environmental changes, and maintenance of long-term reproductive viability (Wilson et al. 2024). Processes such as inbreeding, genetic drift, and the intensive use of a limited number of breeding stock can compromise the adaptive potential of populations and increase the risk of inbreeding depression (Gutiérrez-Reinoso et al. 2022; Speak et al. 2024). In this regard, population genetic characterization enables the identification of patterns of relatedness, gene flow, and genetic differentiation, which are essential for designing management and conservation strategies (Hu et al. 2023).

The use of genomic tools based on single nucleotide polymorphisms (SNPs) has transformed the study of population genetics in domestic species. Due to their high density, uniform distribution across the genome, SNPs allow for high-resolution assessment of genetic diversity, estimation of inbreeding levels, and characterization of population structure at both intra- and inter-breed scales (Yang et al. 2013; Ogunbawo et al. 2024). In Zebu breeds, these approaches are particularly relevant due to their complex domestication history, geographic dispersal, and adaptation to contrasting tropical environments (Dixit et al. 2021).

Despite the productive and economic importance of Gir cattle in Mexico, there is limited information on their genetic diversity, inbreeding levels, and population relationships based on genomic data. This lack of information hinders the assessment of the population’s current genetic status and limits the implementation of breeding and conservation strategies based on genomic evidence.

Therefore, the objective of this study was to evaluate genetic diversity, estimate genomic inbreeding, and characterize the population structure of Mexican Gir cattle using genome-wide SNP markers. We hypothesized that Mexican Gir cattle retain high genetic diversity, exhibit low recent inbreeding, and maintain strong genomic affinity with South Asian Gir populations. The results will provide a solid genomic basis for sustainable management, conservation of genetic variability, and strengthening of genetic improvement programs for this breed in Mexico.

## Materials and methods

### Ethical approval statement

The genomic data used in this study were obtained from pre-collected hair samples, and no direct interventions, experimental procedures, or live animal handling were performed by the authors. Consequently, specific institutional ethical approval was not required for this study.

### Animals, genotyping, and quality control

Two sets of SNPs were utilized in this study. The first set was used for within-breed genetic analysis and included only Gir Zebu genotypes. Hair follicle samples from Gir Zebu cattle were collected by the AMCC. The sampled population comprised 226 purebred animals from three farms located in tropical regions of Mexico (55, 115, and 56 animals). The sample consisted of adult breeding females. Notably, these animals belong to the 1064 active cows evaluated in the most recent national genetic evaluation for the Gir breed (AMCC 2021). These three farms are among the only nine herds that participate in the national genetic evaluation for the Gir breed. Furthermore, these herds are connected using Artificial Insemination (AI) and related sires. Individuals within each farm were randomly selected, ensuring the exclusion of parent-offspring relationships based on pedigree records. Only purebred animals, as confirmed by pedigree and genomic records, were included. To ensure maximum reliability, internal ADMIXTURE and PCA analyses which included reference taurine and indicine breeds as quality control filter were used. Individual ancestry proportions to identify any hidden crossbreeding was verified.

Hair samples were genotyped using the GGP Indicus 50K chip aligned to the ARS-UCD1.2 assembly, which contains approximately 54,709 uniformly distributed informative SNPs with a median spacing of 50.4 kb.

Quality control was done using *PLINK v1.9*, excluding mitochondrial, X and Y chromosomes for further analysis (2656 SNPs were removed). SNPs with genotyping call rate less than 0.90 were also removed (1080). In addition, removal of SNPs that were monomorphic and having minor allele frequency (MAF) lower than 0.01, was done (7400). Animals with a call rate lower than 0.95 (nine) and with relationships > 0.25 were also removed (29) using the—rel-cutoff parameter in *PLINK*. Data was filtered for linkage disequilibrium (LD) with –indep-pairwise 50 5 0.5, call which removes SNPs with squared correlation coefficient between two alleles of two SNPs (r^2^) higher than 0.5 within 50-SNP sliding windows, with each window overlapping by 5 SNPs using *PLINK v1.9* (17,835 SNPs removed). Therefore, after performing quality control, 28,394 SNPs and 174 animals were kept for *HO*, *HE*, $F_{IS}$ and $F_{\mathrm{GRM}}$ estimations. A separate quality control approach was performed for runs of homozygosity (ROH), where LD based pruning and MAF threshold were not applied. A set of 174 animals and 51055 SNPs were retained for use in ROH estimations.

For analysis involving comparisons among breeds, a random sample of 20 animals was selected from the 226 Mexican Gir cattle available in this study to minimize potential biases associated to unequal sample sizes among populations. These animals were analyzed together with 1543 individuals representing 134 cattle breeds obtained from a public repository (Decker et al. 2014). Details on these animals are presented in Table S1. These animals were genotyped using the Illumina BovineSNP50 BeadChip array and the UMD3.0 assembly. The authors applied a call rate threshold of 90% and removed monomorphic SNPs, resulting in 43,043 available SNPs (Decker et al. 2014).

To ensure compatibility between genotyping arrays based on different bovine genome assemblies, genomic coordinates from the Decker et al. dataset, originally annotated according to the UMD3.1 assembly (bosTau6), were converted to the ARS-UCD1.2 assembly (bosTau9) using the UCSC LiftOver tool and the corresponding chain file. Of the 43,043 SNPs initially available, 42,889 were successfully mapped to the new assembly, whereas 154 SNPs that failed coordinate conversion were excluded. Subsequently, 25 SNPs with duplicated genomic coordinates were removed. Marker harmonization between datasets was performed based on chromosome and physical position. Strand inconsistencies were identified during dataset merging and resolved by allele flipping using PLINK, affecting 3,279 SNPs.

After marker harmonization, a total of 9,091 SNPs shared between arrays were retained for across-breed analyses. These markers were distributed across all 29 bovine autosomes. The average inter-marker distance was consistent among chromosomes (average of 270 kb). A summary of SNP distribution across chromosomes is provided in Table S2.

### Within-breed genetic diversity

Observed (HO) and expected (HE) heterozygosity were estimated, and the inbreeding coefficient was calculated using three different methods: 1) $F_{IS}$, based on the difference between observed and expected homozygosity; 2) $F_{\mathrm{GRM}}$, calculated from the diagonal elements of the genomic relationship matrix; and 3) $F_{\mathrm{ROH}}$, based on runs of homozygosity (ROH). HO, HE, and $F_{IS}$ were calculated using PLINK (Purcell et al. 2007). The inbreeding coefficient $F_{\mathrm{GRM}}$ was estimated using the AGHmatrix package in R, following the method of VanRaden (2008).

The ROH were computed for each animal using the following parameters: (1) minimum ROH length of 1 Mb; (2) a minimum of 20 consecutive homozygous SNPs per ROH; (3) a maximum of one heterozygous SNP allowed per ROH; (4) a maximum inter-SNP gap of 1,000 kb; and (5) a maximum of one missing genotype allowed per ROH. ROH were calculated using the R package DetectRUNS (Biscarini et al. 2020). The minimum SNP density was set at one SNP per 1,000 kb, and the maximum gap between consecutive SNPs was 1 Mb.

$F_{\mathrm{ROH}}$ was computed according to McQuillan et al. (2008) as:


$$F_{\mathrm{ROH}}=\frac{L_{\mathrm{ROH}}}{L_{\mathrm{AUT}}},$$

where $L_{\mathrm{ROH}}$ is the total length of all the ROH detected in the animal’s autosomes, $L_{\mathrm{AUT}}$ is the total length of the autosomal genome covered by the SNP markers. The ROHs in the population were categorized into five length classes (1–2 Mb, 2–4 Mb, 4–8 Mb, 8–16 Mb, and > 16 Mb) to compare the distribution of $F_{\mathrm{ROH}}$ among them.

### Across-breed genetic diversity

To assess between-breed genetic diversity, multidimensional scaling (MDS) was used to describe genetic differentiation between Gir Zebu cattle and other populations. MDS was performed on the merged dataset, using only autosomal SNPs, with PLINK. The top two dimensions were plotted to visualize the relationships between Gir Zebu cattle and the other 134 breeds.

To compare with more specific breeds, we generated a subset of the reference dataset that included Mexican Gir and other Zebu breeds. Pairwise F_ST_ values between Gir and other Zebu breeds were calculated using the—F_ST_ option in PLINK (Purcell et al. 2007). To estimate genotypic similarity among individuals, we used the—distance option of PLINK, which is based on identity by state (IBS) to generate a square matrix reflecting the proportion of alleles shared between pairs of individuals. This matrix was visualized as a heatmap using the pheatmap package in R.

To assess the population structure of Gir cattle relative to other breeds, we analyzed genotypes from 28 cattle breeds using autosomal SNPs. Population ancestry ratios were estimated using ADMIXTURE v1.3.0 (Alexander et al. 2009), which employs a maximum-likelihood approach to infer individual ancestry from multilocus SNP data. The analysis was performed on the pruned dataset to reduce linkage disequilibrium and ensure high-quality genotypes. We tested K values ranging from 2 to 30, and the optimal number of clusters was determined as the K value with the lowest cross-validation error. The resulting ancestry ratios were visualized using bar charts to illustrate the genetic contributions of each inferred ancestral population to individuals and breeds.

## Results

### Within-breed genetic diversity

The average HO and HE with the first SNP dataset were 0.40 ± 0.013 and 0.38 ± 0.001, respectively. The $F_{IS}$, $F_{\mathrm{GRM}}$, and $F_{\mathrm{ROH}}$ were −0.04, -0.02, and 0.07, respectively, indicating a low overall level of inbreeding in the Mexican Gir population. Summary statistics of ROH and $F_{\mathrm{ROH}}$ in different length classes (1–2, 2–4, 4–8, 8–16, and > 16 Mb) are reported in Table 1.

**Table 1:** Amount, mean length, number of SNPs, and inbreeding per runs of homozygosity, in different length classes for animals carrying ROH.

ROH^1^length class (Mb)	n	Mean length (Mb ± SD)	Mean SNP^2^ ± SD	$F_{\mathrm{ROH}}$ ^3^ ± SD
**1–2**	8338	1.337 ± 0.248	26.622 ± 6.561	0.026 ± 0.004
**2–4**	1601	2.723 ± 0.565	54.974 ± 16.091	0.010 ± 0.003
**4–8**	668	5.521 ± 1.12	113.813 ± 27.103	0.009 ± 0.005
**8–16**	325	10.841 ± 2.133	221.618 ± 46.543	0.011 ± 0.007
**>16**	95	24.373 ± 8.075	491.842 ± 162.383	0.013 ± 0.007

1 ROH= Runs of homozygosity.

2 SNP= Single nucleotide polymorphism.

3 $F_{\mathrm{ROH}}$ =Inbreeding calculated by runs of homozygosity.

The Spearman correlations among the different inbreeding coefficients and their significance level are presented in Table 2. The highest correlations were observed between $F_{\mathrm{ROH}}$ and the coefficients based on long ROH segments, whereas lower correlations were found for coefficients calculated from shorter ROH classes (1–2 Mb and 2–4 Mb). This pattern suggests that long ROH contribute more substantially to the overall genomic inbreeding estimate than short ROH segments. $F_{\mathrm{ROH}}$ also had a significant and high correlation with $F_{IS}$.

**Table 2:** Spearman’s correlations (above diagonal) among Hardy-Weinberg equilibrium ($F_{IS}$) genomic relationship matrix ($F_{\mathrm{GRM}}$), and runs of homozygosity based ($F_{\mathrm{ROH}}$) inbreeding coefficients. Significance values are reported below the diagonal.

	$F_{\mathrm{ROH}\;1-2\;\mathrm{Mb}}$	$F_{\mathrm{ROH}\;2-4\;\mathrm{Mb}}$	$F_{\mathrm{ROH}\;4-8\;\mathrm{Mb}}$	$F_{\mathrm{ROH}\;8-16\;\mathrm{Mb}}$	$F_{\mathrm{ROH}>16\;\mathrm{Mb}}$	$F_{\mathrm{ROH}\;\mathrm{Total}}$	$F_{\mathrm{IS}}$	$F_{\mathrm{GRM}}$
$F_{\mathrm{ROH}\;1-2\;\mathrm{Mb}}$	1	0.01	0.1	−0.21	−0.1	0.23	−0.08	0.06
$F_{\mathrm{ROH}\;2-4\;\mathrm{Mb}}$	NS	1	−0.01	0.03	0.21	0.25	0.15	0.12
$F_{\mathrm{ROH}\;4-8\;\mathrm{Mb}}$	NS	NS	1	0.08	0.06	0.54	0.32	0.12
$F_{\mathrm{ROH}\;8-16\;\mathrm{Mb}}$	*P* < 0.05	NS	NS	1	0.22	0.5	0.46	0.25
$F_{\mathrm{ROH}>16\;\mathrm{Mb}}$	NS	NS	NS	NS	1	0.6	0.44	0.23
$F_{\mathrm{ROH}\;\mathrm{Total}}$	*P* < 0.01	*P* < 0.001	*P* < 0.001	*P* < 0.001	*P* < 0.001	1	0.6	0.23
$F_{\mathrm{IS}}$	NS	*P* < 0.05	*P* < 0.001	*P* < 0.001	*P* < 0.001	*P* < 0.001	1	0.12
$F_{\mathrm{GRM}}$	NS	NS	NS	*P* < 0.01	NS	*P* < 0.01	NS	1

### Across-breed genetic diversity

**Figure 1:**
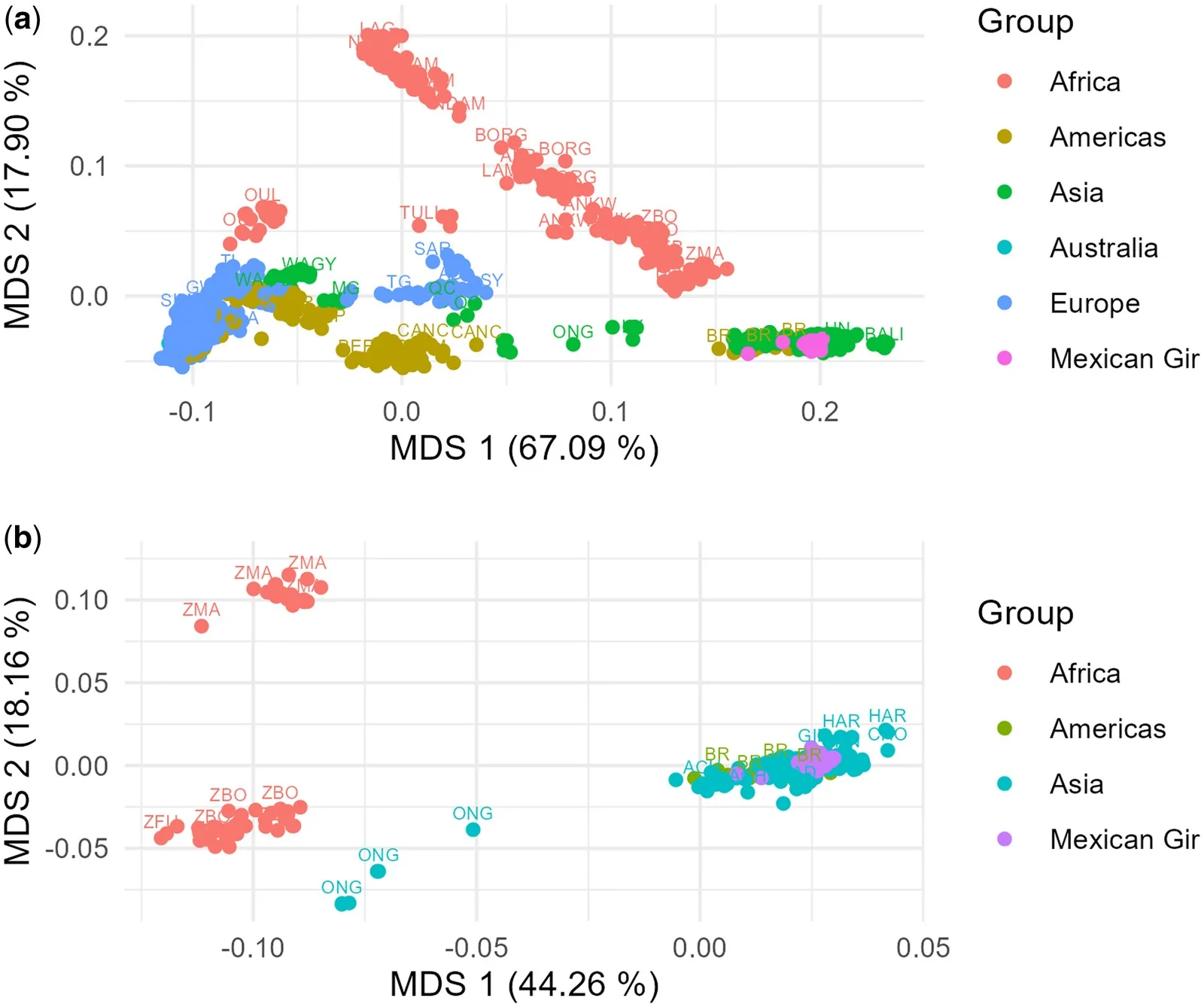
MDS Plots of 134 worldwide cattle breeds (a), only zebu breeds (b), and the mexican gir breed using 9,091 SNPs spread across the genome.

The MDS analysis showed that Mexican Gir Zebu cattle clustered closely with Asian Zebu breeds, especially Gir animals sampled from India and Pakistan (Fig. 1). In Fig. 1a, MDS dimension 1 differentiated *Bos taurus*, *Bos indicus*, and crossbreed cattle. In Fig. 1b, MDS separated African breeds from those of American and Asian origin, with Mexican Gir positioned within the Asian group.

**Figure 2:**
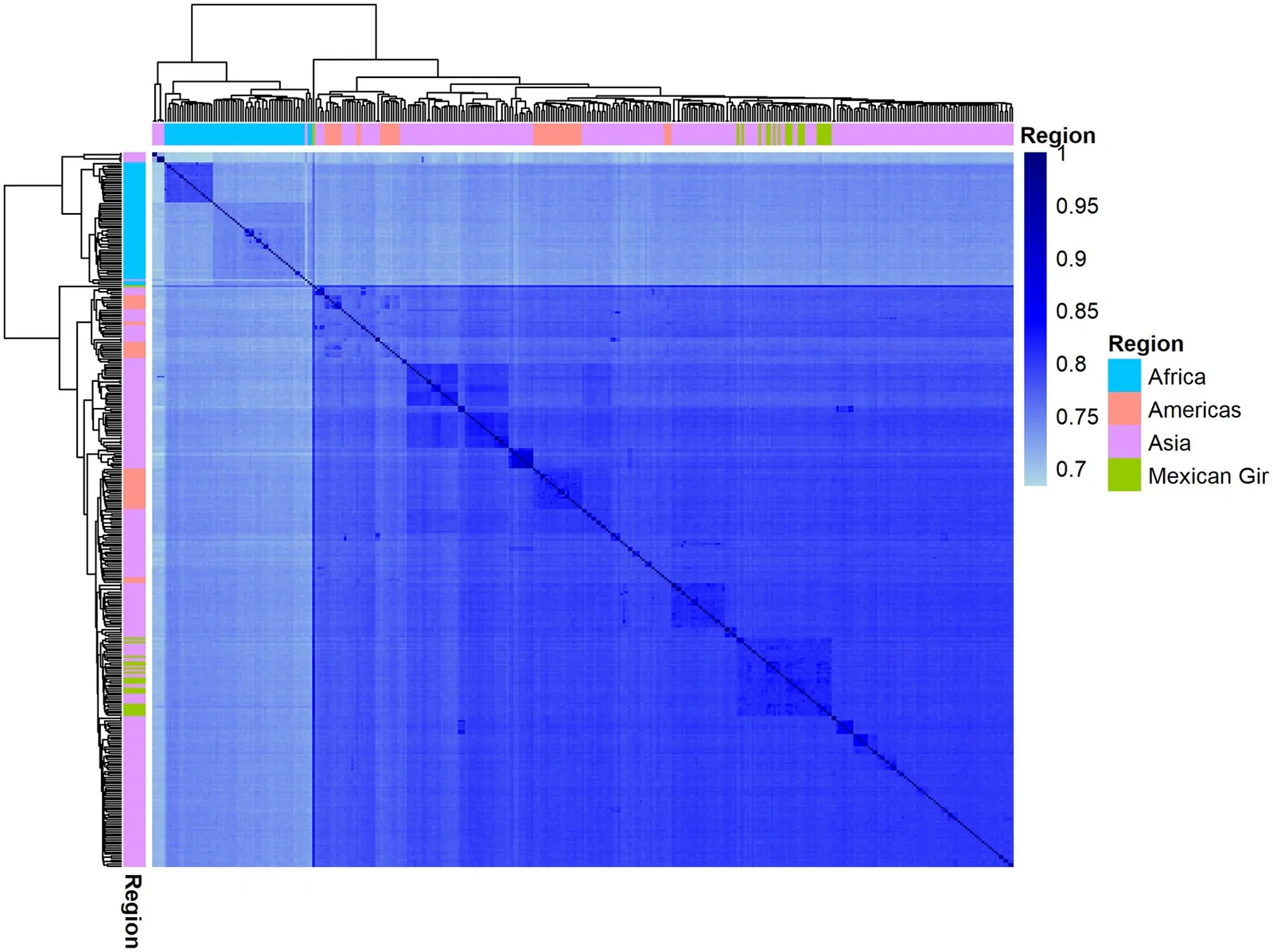
Heatmap of the identical by state (IBS) matrix for zebu cattle breeds.

The F_ST_ values between Mexican Gir and the other Zebu breeds are presented in Table 3. The lowest values were observed with Gir (from India), Red Sindhi, Lohani, Rojhan, and Tharparkar breeds, suggesting a close genetic affinity between Mexican Gir and their South Asian ancestors. The genotypic similarity matrix is shown in Fig. 2, where Mexican Gir displayed higher similarity with other Gir populations and Asian breeds than unrelated Zebu breeds.

**Table 3:** Average fixation index (F_ST_) and identity by state (IBS) values between the mexican gir and other zebu breeds worldwide.

Breed	Geographic origin	F_ST_	IBS
**Gir**	Gujerat, India	0.005	0.812
**Red Sindhi**	Sindh, Pakistan	0.049	0.796
**Lohani**	Northwest Pakistan	0.050	0.795
**Rojhan**	Punjab, Pakistan	0.052	0.796
**Tharparkar**	Southeast Sindh, Pakistan	0.054	0.795
**Dhanni**	Punjab, Pakistan	0.055	0.797
**Gabrali**	Khyber Pakhtun Khwa, Pakistan	0.056	0.786
**Dajal**	Punjab, Pakistan	0.058	0.797
**Hissar**	Punjab, Pakistan	0.058	0.796
**Brahman**	Gulf Coast, United States	0.059	0.780
**Ongole**	Andhra Pradesh, India	0.068	0.769
**Sahiwal**	Punjab, Pakistan	0.073	0.795
**Achai**	Khyber Pakhtun Khwa, Pakistan	0.074	0.781
**Kankraj**	North Gujarat, India	0.082	0.799
**Nelore**	Brazil	0.084	0.792
**Bhagnari**	Kaochi, Kalat, and Balochistan, Pakistan	0.086	0.788
**Guzerat**	Guzarat, India	0.095	0.793
**Cholistani**	from Cholistan, Punjab, Pakistan	0.095	0.796
**Aceh**	Sumatra, Indonesia	0.099	0.793
**Brebes**	Indonesia	0.109	0.784
**Bororo Zebu**	Chad	0.120	0.736
**Fulani zebu**	Benin	0.139	0.733
**Hainan**	Hainan Province, China	0.148	0.782
**Madura**	Madura Island, Indonesia	0.153	0.775
**Hariana**	Haryana plains, India	0.155	0.792
**Pesisir**	Indonesia	0.159	0.785
**Zebu of Madagascar**	Madagascar	0.177	0.742

**Figure 3:**
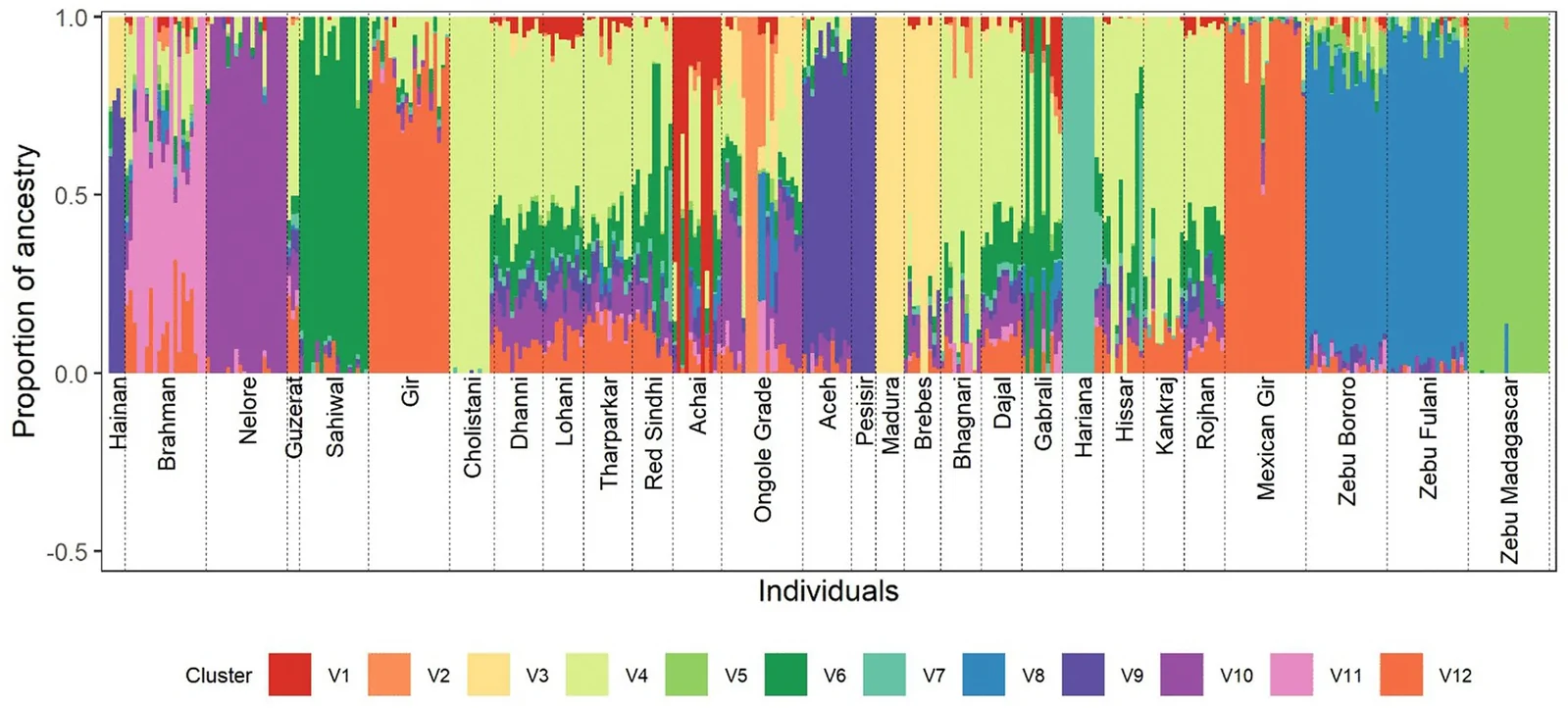
Admixture plot (K = 12) of zebu breeds, and the mexican gir breed.

Population structure analysis using ADMIXTURE (355 individuals from 28 breeds/populations; SNPs pruned for linkage disequilibrium) is shown in Fig. 3. The value with the lowest cross-validation error is K = 12 (Table S3 and Fig. S1). Under this model, K = 12 means that Gir population was divided into 12 components, from V1 to V12, showing a predominant assignment to the V12 component (mean = 0.930, SD = 0.118; range = 0.501–0.993). The Indian Gir showed the same principal component (V12), with a lower average proportion (mean = 0.829, SD = 0.104; range = 0.647–0.991). Overall, both populations shared the dominant V12 component, and the Mexican Gir showed a higher proportion of assignment to it than the Indian Gir.

For comparison, other Indicus populations showed distinct dominant components and varying degrees of concentration in a single component: Nelore was primarily assigned to V10 (mean = 0.936, SD = 0.098), while Brahman was dominated by V11 (mean = 0.609, SD = 0.328) with additional contributions from V4 (mean = 0.134) and V12 (mean = 0.117). On the Indian subcontinent, several breeds (eg Cholistani and Kankraj) exhibited a predominance of V4 (Cholistani mean = 0.953; Kankraj mean = 0.779), while Sahiwal and Hariana showed a predominance of V6 and V7, respectively (Sahiwal mean = 0.915; Hariana mean = 0.840). In Insulindia/SE Asia, Madura and Brebes were mainly associated with V3 (Madura ≈ 1.000; Brebes mean = 0.778, SD = 0.150), while Aceh, Pesisir, and Hainan showed a predominance of V9 (Aceh mean = 0.811; Pesisir ≈ 1.000; Hainan = 0.722); in Africa, Zebu Fulani and Zebu from Madagascar were mainly assigned to V8 and V5, respectively (Zebu Fulani mean = 0.912, SD = 0.048; Madagascar mean = 0.991, SD = 0.019). Finally, Ongole Grade showed a pattern distributed among several components (V10 = 0.244; V2 = 0.208; V3 = 0.176).

## Discussion

### Within-breed genetic diversity

While Mexico possesses a vast tropical cattle population, the vast majority of these animals consist of crossbred individuals rather than purebred stock. The purebred Mexican Gir population is highly consolidated; for instance, the national genetic evaluation program recently recorded only 1,064 active cows evaluated for EPDs and approximately 88,000 historical pedigree records (CCG 2025). Consequently, the 226 purebred animals sampled in this study from three primary nucleus herds represent more than 20% of the actively evaluated contemporary purebred population.

The values for observed and expected heterozygosity (HO = 0.40; HE = 0.38) in Mexican Gir cattle indicate a remarkable level of genetic diversity, comparable to international reports for other Gir populations (HO: 0.27–0.41; HE: 0.27–0.38). These values fall within the upper range reported for Gir populations in Brazil and India and are comparable to those of other indicine breeds genotyped using medium-density SNP panels (Dixit et al. 2021; Corredor et al. 2023; Braga et al. 2025). The slight excess of heterozygosity observed over that expected (HO > HE) suggests that the analyzed population has maintained a broad genetic base, despite historical processes of introduction and expansion outside its region of origin. The slight excess of heterozygosity observed compared to the expected value (HO > HE) is particularly relevant, as it contrasts with the heterozygote deficit typically observed in populations subjected to recent inbreeding or severe bottlenecks (Howard et al. 2017). This excess can be interpreted as evidence of an open population structure, possibly associated with the mixing of founder lineages, genetic exchange between herds, or reproductive management practices that avoid close inbreeding. Similar patterns have been described in other cattle breeds with a history of continuous gene flow (Decker et al. 2014; Pitt et al. 2019).

Inbreeding coefficients estimated using different genomic approaches reinforce these conclusions. The value $F_{IS}=-0.04$ indicates an excess of heterozygotes relative to Hardy–Weinberg expectations. This result suggests lower-than-expected homozygosity under the reference allele frequencies used and may reflect the effects of admixture, selection, or other demographic processes (Halvoník et al. 2024).

Consistently, $F_{\mathrm{GRM}}$ based on genomic relationships summarizes the deviation of observed homozygosity in the population relative to the reference allele frequencies; a value of −0.02 indicates that the population has 2% less homozygosity than expected, or equivalently, slightly more heterozygosity than expected (VanRaden 2008). However, negative values of $F_{IS}$ and $F_{\mathrm{GRM}}$ should not be interpreted as evidence for the complete absence of inbreeding, but rather as an indication that the population exhibits lower-than-expected homozygosity according to these metrics.

The estimated $F_{\mathrm{ROH}}=0.07$ indicates that approximately 7% of the genome is in homozygous regions; this value is lower than estimates reported in bovine populations subjected to intensive selection or with reduced effective population sizes (Mastrangelo et al. 2016; Pilon et al. 2021). Furthermore, the predominance of short ROHs observed in this population is indicative of ancient inbreeding or historical demographic events. In contrast, the scarcity of long ROHs suggests that recent inbreeding resulting from matings between close relatives has been minimal (Kirin et al. 2010).

The correlations observed between the different inbreeding coefficients provide information about the consistency of the genomic estimators used in this study. In particular, the high correlation between $F_{\mathrm{ROH}}$ and $F_{\mathrm{GRM}}$, as well as between $F_{\mathrm{ROH}}$ and $F_{IS}$, indicates agreement between approaches based on homozygosity segments and those derived from genome-wide marker information. This consistency suggests that the different estimators capture similar patterns of genomic variation within the Mexican Gir population. The predominance of short ROH segments observed in Table 1 provides additional insight into the temporal origin of inbreeding. Because short ROH are generally associated with more distant common ancestors, their high frequency suggests that much of the detected autozygosity is likely attributable to historical rather than recent inbreeding.

Taken together, the agreement among the three inbreeding estimators ($F_{IS}=-0.04$, $F_{\mathrm{GRM}}=-0.02$, and $F_{\mathrm{ROH}}=0.07$) and the dominance of short ROH values suggest that the Mexican Gir exhibits a low overall level of inbreeding. This pattern is comparable to that observed in other Gir populations and Zebu breeds with histories of geographic expansion and relatively diverse reproductive management (Peripolli et al. 2018), and suggests that the implemented selection and breeding programs have contributed to the breed’s genetic integrity.

### Across-breed genetic diversity

The MDS analysis (Fig. 1a), based on 134 cattle breeds, show the breeds with genetic relationships close to Mexican Gir. The first dimension consistently separated *Bos taurus*, *Bos indicus*, and hybrid breeds, a pattern widely documented in genome-wide studies of bovine diversity (Gebrehiwot et al. 2020; Ward et al. 2022). The placement of the Mexican Gir within the *Bos indicus* cluster suggests that this population maintains its indicus genetic identity, without evidence of significant taurine introgression, despite its long history of management in American production systems where breeds of different origins coexist (Decker et al. 2014; O’Brien et al. 2015).

Restricting the MDS analysis exclusively to Zebu breeds (Fig. 1b) revealed further differentiation along the first dimension, clearly separating African Zebu from those of Asian and American origin. In this context, the Mexican Gir clustered closely with Asian Gir, consistently, reflecting the historical route of Gir introduction from Asia to the Americas (AMCC 2021; CCG 2025). If the second dataset from public repository had been included Brazilian Gir (Fig. 3), possibly Mexican Gir could be clustered closely with them; therefore, in our results Mexican Gir was more related to Asian Gir. The observed genetic distance from African Zebu suggests that they have not substantially contributed to the Mexican Gir’s gene pool, reinforcing the hypothesis of a predominantly South Asian origin (Chen et al. 2023; Yadav et al. 2025).

This pattern is corroborated by F_ST_-based genetic differentiation analyses. The extremely low F_ST_ values between the Mexican Gir and the Indian Gir (F_ST_ = 0.005), as well as with breeds such as Red Sindhi, Lohani, Rojhan, and Tharparkar (F_ST_ ≈ 0.05), indicated a close genetic relationship and a shared ancestral origin (Strucken et al. 2021; Hall 2022). In contrast, the higher values observed with African and Southeast Asian Zebus (eg Madagascar and Benin) reflect greater genetic divergence, consistent with an earlier geographic and demographic separation (Decker et al. 2014; Magnier et al. 2022; Chen et al. 2023)_._ The increasing F_ST_ gradient with geographic distance supports a differentiation model primarily driven by the evolutionary history and dispersal routes of the Zebu (van Strien et al. 2015).

Genotypic similarity analysis based on IBS reinforced these results using an independent metric. The Mexican Gir showed high genomic similarity to the Indian Gir and other Asian Zebus, while exhibiting considerably lower similarity values to African Zebus. The similarity between the patterns observed using F_ST_ and IBS suggested the inferred genetic relatedness and minimized the likelihood that the results are due to methodological bias (Purcell et al. 2007; Chen et al. 2023).

The population structure analysis using ADMIXTURE indicated, based on the optimal number of clusters (K), that the breed’s characteristic genomic profile is conserved in the sampled population in Mexico and maintains a clear correspondence with the pattern observed in its region of origin. Consistently, the population structure analysis using ADMIXTURE showed shared genetic components with Asian Zebus; furthermore, the findings showed that recent admixture with other breeds suggests that, despite its establishment outside its area of origin, it has maintained a stable and coherent genetic structure, consistent with relatively controlled reproductive management and limited exogenous introgression (Campos et al. 2017; Verardo et al. 2021).

Taken together, the results derived from MDS, F_ST_, IBS and ADMIXTURE converge on the same biological pattern: this sample from the Mexican Gir population retains its Indicine genetic identity, maintains a strong affinity with Zebu breeds from South Asia and constitutes a genetically well-defined population, whose differentiation from other Zebu breeds mainly reflects historical processes of dispersal and adaptation, rather than recent events of genetic mixing.

## Conclusions

This study provides a genomic characterization of Mexican Gir cattle, offering evidence on their genetic diversity, inbreeding levels, and population relationships within a global context. The results obtained from high-density SNP markers suggest that the Gir population in Mexico maintains high genetic variability, comparable to that reported for Gir populations in other countries. The various estimators of genomic inbreeding ($F_{IS}$, $F_{\mathrm{GRM}}$, and $F_{\mathrm{ROH}}$) agreed in indicating a low level of recent inbreeding, with a predominant contribution from short homozygosity ranges, suggesting that the observed inbreeding has a primarily historical origin and does not reflect current reproductive practices based on mating between close relatives. The agreement between the ROH-based and genomic relationship-based approaches suggests that the use of genomic tools is appropriate methods for monitoring inbreeding in bovine populations. Population structure and genetic relationship analyses (MDS, F_ST_, IBS_,_ and ADMIXTURE) showed that the Mexican Gir clusters within the Bos indicus lineage, with no evidence of significant taurine introgression. Furthermore, a genetic affinity was observed with Zebu breeds from South Asia, particularly the Indian Gir and related breeds from Pakistan, which is consistent with the documented history of the breed’s introduction and dispersal to the Americas. Genetic differentiation from African and other zebu breeds supports a divergence model primarily associated with historical and geographic processes. These results suggest that the Mexican Gir cattle population is genetically well-defined and has low recent inbreeding, providing a favorable foundation for strengthening genetic improvement programs focused on productivity, adaptation to the tropics, and sustainability. The study underscores the importance of continuous genomic monitoring, balanced use of sires, and the strategic introduction of genetic material as key tools for preserving genetic diversity and ensuring the long-term viability of the breed in Mexico.

## Supplementary data

Supplementary data is available at *Translational Animal Science* online.

## Supplementary Material

txag094_Supplementary_Data

## Data Availability

The genotype data, filtered SNP list, scripts, and breed metadata that supports the findings of this study are available from the corresponding author upon reasonable request.

## List of abbreviations

AGHmatrix: R package for computing genomic relationship matrices
AMCC: Asociación Mexicana de Criadores de Cebú
ARS-UCD1.2: Bos taurus reference genome assembly version 1.2
CCG: Criadores de Gyr (México)
F_GRM_: Inbreeding coefficient derived from the genomic relationship matrix
F_IS_: Inbreeding coefficient based on deviation from Hardy–Weinberg equilibrium
F_ROH_: Inbreeding coefficient based on runs of homozygosity
F_ST_: Fixation index (measure of genetic differentiation among populations)
HE: Expected heterozygosity
HO: Observed heterozygosity
IBS: Identity by state
K: Number of ancestral clusters inferred in population structure analysis
kb: Kilobase
LD: Linkage disequilibrium
Mb: Megabase
MDS: Multidimensional scaling
PLINK: Whole-genome association analysis toolset
r^2^: Squared correlation coefficient used in linkage disequilibrium pruning
ROH: Runs of homozygosity
SD: Standard deviation
SNP: Single nucleotide polymorphism
UMD3.0: University of Maryland bovine genome assembly version 3.0
