# Supplementary material for: Genetic diversity and population structure of Mexican Gir cattle using genome-wide SNP data
Source: Transl Anim Sci. 2026 Jul 17;10:txag094. doi: 10.1093/tas/txag094 (PMC13588704; doi:10.1093/tas/txag094)
Supplement: Supplementary file 1 [file txag094_supplementary_data.docx]

Table S1. Breeds and number of samples included in the analysis.

| Breed | No. Samples | (Sub)Species | Continent | Geographic Origin |
| --- | --- | --- | --- | --- |
| Gir | 226 | Bos t. indicus | Americas | Mexico |
| Bali | 20 | Bos javanicus | Asia | Bali, Indonesia |
| Zebu Fulani | 20 | Bos t. indicus | Africa | Benin |
| Zebu Bororo | 20 | Bos t. indicus | Africa | Chad |
| Zebu from Madagascar | 20 | Bos t. indicus | Africa | Madagascar |
| Nelore | 20 | Bos t. indicus | Americas | Brazil |
| Brahman | 20 | Bos t. indicus | Americas | Gulf Coast, United States |
| Ongole Grade | 20 | Bos t. indicus | Asia | Andhra Pradesh, India |
| Achai | 12 | Bos t. indicus | Asia | Khyber Pakhtun Khwa, Pakistan |
| Red Sindhi | 10 | Bos t. indicus | Asia | Sindh, Pakistan |
| Cholistani | 11 | Bos t. indicus | Asia | Cholistan Desert, Punjab, Pakistan |
| Gir | 20 | Bos t. indicus | Asia | Gujerat, India |
| Guzerat | 3 | Bos t. indicus | Asia | Guzarat, India |
| Hariana | 10 | Bos t. indicus | Asia | Haryana plains, India |
| Brebes | 9 | Bos t. indicus | Asia | Indonesia |
| Pesisir | 6 | Bos t. indicus | Asia | Indonesia |
| Dajal | 10 | Bos t. indicus | Asia | Punjab, Pakistan |
| Bhagnari | 10 | Bos t. indicus | Asia | Kaochi, Kalat, and Baluchistan, Pakistan |
| Gabrali | 10 | Bos t. indicus | Asia | Khyber Pakhtun Khwa, Pakistan |
| Hainan | 4 | Bos t. indicus | Asia | Hainan Province, China |
| Madura | 7 | Bos t. indicus | Asia | Madura Island, Indonesia |
| Kankraj | 10 | Bos t. indicus | Asia | North Gujerat, India |
| Lohani | 10 | Bos t. indicus | Asia | Northwest Pakistan |
| Dhanni | 12 | Bos t. indicus | Asia | Punjab, Pakistan |
| Hissar | 10 | Bos t. indicus | Asia | Punjab, Pakistan |
| Sahiwal | 17 | Bos t. indicus | Asia | Punjab, Pakistan |
| Tharparkar | 12 | Bos t. indicus | Asia | Southeast Sindh, Pakistan |
| Rojhan | 10 | Bos t. indicus | Asia | Punjab, Pakistan |
| Aceh | 12 | Bos t. indicus | Asia | Sumatra, Indonesia |
| Lagune | 20 | Bos t. taurus | Africa | Benin |
| Baoule | 20 | Bos t. taurus | Africa | Burkina Faso |
| Kuri | 20 | Bos t. taurus | Africa | Chad |
| N'Dama | 20 | Bos t. taurus | Africa | Ivory Coast, Africa |
| Oulmès Zaer | 19 | Bos t. taurus | Africa | Morocco |
| N'Dama | 14 | Bos t. taurus | Africa | Southeast Burkina Faso |
| N'Dama | 17 | Bos t. taurus | Africa | Southwest Burkina Faso |
| Somba | 20 | Bos t. taurus | Africa | Togo |
| Romosinuano | 8 | Bos t. taurus | Americas | Columbia |
| Florida Cracker | 9 | Bos t. taurus | Americas | Florida, United States |
| Corriente | 5 | Bos t. taurus | Americas | Sonora, Mexico |
| Texas Longhorn | 20 | Bos t. taurus | Americas | Texas, United States |
| Brown Swiss | 20 | Bos t. taurus | Americas | United States |
| Red Angus | 19 | Bos t. taurus | Americas | United States |
| Senepol | 19 | Bos t. taurus | Americas | United States Virgin Islands |
| Wagyu | 12 | Bos t. taurus | Asia | Japan |
| Hanwoo | 8 | Bos t. taurus | Asia | Korea |
| Mongolian | 5 | Bos t. taurus | Asia | Mongolia1 |
| Murray Grey | 4 | Bos t. taurus | Australia | Australia |
| Angus | 20 | Bos t. taurus | Europe | Aberdeenshire, Scotland |
| Tarentaise | 5 | Bos t. taurus | Europe | Alpine Massif-Central of southeastern France |
| Pinzgauer | 5 | Bos t. taurus | Europe | Austria |
| Belgian Blue | 4 | Bos t. taurus | Europe | Belgium |
| Simmental | 20 | Bos t. taurus | Europe | Bern, Switzerland |
| Simmentaler | 4 | Bos t. taurus | Europe | Bern, Switzerland |
| Maine-Anjou | 20 | Bos t. taurus | Europe | Brittany, France |
| Rendena | 3 | Bos t. taurus | Europe | Central Alps |
| Gelbvieh | 20 | Bos t. taurus | Europe | Central Germany |
| Berrenda en Negro | 5 | Bos t. taurus | Europe | Ciudad Real, Jaen, Cordoba, Sevilla, and Huelva, Spain |
| Berrenda en Colorado | 5 | Bos t. taurus | Europe | Cordoba, Sevilla, Huelva, and Cadiz, Spain |
| Devon | 4 | Bos t. taurus | Europe | Devon, England |
| Romagnola | 20 | Bos t. taurus | Europe | Emilia, Italy |
| Beef Shorthorn | 17 | Bos t. taurus | Europe | England |
| Lincoln Red | 9 | Bos t. taurus | Europe | England |
| Milking Shorthorn | 9 | Bos t. taurus | Europe | England |
| Montbeliard | 20 | Bos t. taurus | Europe | France |
| Normande | 20 | Bos t. taurus | Europe | France |
| Guernsey | 20 | Bos t. taurus | Europe | Guernsey Island |
| Holstein | 20 | Bos t. taurus | Europe | Holland |
| Dexter | 4 | Bos t. taurus | Europe | Ireland |
| Kerry | 3 | Bos t. taurus | Europe | Ireland |
| Marchigiana | 2 | Bos t. taurus | Europe | Italy |
| Jersey | 20 | Bos t. taurus | Europe | Jersey Island |
| Lithuanian Light Grey | 2 | Bos t. taurus | Europe | Lithuania |
| Lithuanian White Backed | 3 | Bos t. taurus | Europe | Lithuania |
| Limousin | 20 | Bos t. taurus | Europe | Massif Central, France |
| Salers | 20 | Bos t. taurus | Europe | Massif Central, France |
| Menorquina | 3 | Bos t. taurus | Europe | Menorca, Spain |
| Mostrenca | 5 | Bos t. taurus | Europe | National Park of Donana, southwestern Spain |
| Groningen Whitehead | 2 | Bos t. taurus | Europe | Netherlands |
| Lakenvelder | 1 | Bos t. taurus | Europe | Netherlands |
| Meuse-Rhine-Ijjsel | 3 | Bos t. taurus | Europe | Netherlands |
| Red Poll | 5 | Bos t. taurus | Europe | Norfolk and Suffolk, England |
| Vosgienne | 20 | Bos t. taurus | Europe | Northeast France |
| Bretonne Black Pied | 18 | Bos t. taurus | Europe | Northwest France |
| French Red Pied Lowland | 20 | Bos t. taurus | Europe | Northwest France |
| Maraichine (Parthenaise) | 19 | Bos t. taurus | Europe | Northwest France |
| Piedmontese | 20 | Bos t. taurus | Europe | Northwest Italy |
| Pirenaica | 5 | Bos t. taurus | Europe | northwest of Spain |
| Norwegian Red | 20 | Bos t. taurus | Europe | Norway |
| Blonde d'Aquitaine | 5 | Bos t. taurus | Europe | Pyrenees, France |
| Morucha | 5 | Bos t. taurus | Europe | Salamanca |
| Charolais | 20 | Bos t. taurus | Europe | Saône-et-Loire, France |
| Belted Galloway | 4 | Bos t. taurus | Europe | Scotland |
| Galloway | 4 | Bos t. taurus | Europe | Scotland |
| Finnish Ayrshire | 18 | Bos t. taurus | Europe | Scotland/Finland |
| Negra Andaluza | 5 | Bos t. taurus | Europe | Sierra Morena Mountains, Cordoba, and Sevilla Spain |
| Cardena Andaluza | 5 | Bos t. taurus | Europe | Sierra Morena, Spain |
| South Devon | 3 | Bos t. taurus | Europe | South England |
| Aubrac | 20 | Bos t. taurus | Europe | South France |
| Sussex | 4 | Bos t. taurus | Europe | Southeast England |
| Abondance | 20 | Bos t. taurus | Europe | Southeast France |
| Tarine | 18 | Bos t. taurus | Europe | Southeast France |
| Gascon | 20 | Bos t. taurus | Europe | Southwest France |
| Retinta | 4 | Bos t. taurus | Europe | Southwest of Spain and bordering Portugal |
| Toro de Lidia | 4 | Bos t. taurus | Europe | Spain |
| Toro de Lidia | 5 | Bos t. taurus | Europe | Spain |
| Braunvieh | 20 | Bos t. taurus | Europe | Switzerland |
| Ehringer | 2 | Bos t. taurus | Europe | Switzerland |
| Anatolian Black | 8 | Bos t. taurus | Europe | Turkey |
| Anatolian Southern Yellow | 8 | Bos t. taurus | Europe | Turkey |
| East Anatolian Red | 8 | Bos t. taurus | Europe | Turkey |
| South Anatolian Red | 8 | Bos t. taurus | Europe | Turkey |
| Turkish Grey | 8 | Bos t. taurus | Europe | Turkey |
| Zavot | 5 | Bos t. taurus | Europe | Turkey |
| Terrana | 5 | Bos t. taurus | Europe | Vasconcades mountainous region of Alava, Spain |
| Hereford | 20 | Bos t. taurus | Europe | Wales |
| Welsh Black | 2 | Bos t. taurus | Europe | Wales |
| White Park | 5 | Bos t. taurus | Europe | Wales |
| Chianina | 9 | Bos t. taurus | Europe | West Central Italy |
| Scottish Highland | 8 | Bos t. taurus | Europe | Western Scottland |
| Longhorn | 3 | Bos t. taurus | Europe | Yorkshire, England |
| Borgou | 20 | Hybrid | Africa | Benin |
| Tuli | 4 | Hybrid | Africa | Botswana |
| Landim | 1 | Hybrid | Africa | East Coast of South Africa |
| Sheko | 17 | Hybrid | Africa | Ethiopia |
| East African Shorthorn Zebu | 20 | Hybrid | Africa | Kenya |
| Ankole-Watusi | 5 | Hybrid | Africa | Ruanda |
| Africander | 4 | Hybrid | Africa | South Africa |
| Boran | 20 | Hybrid | Africa | southern Ethiopia |
| Canchim | 20 | Hybrid | Americas | Brazil |
| Beefalo | 1 | Hybrid | Americas | Northwest United States |
| Beefmaster | 20 | Hybrid | Americas | Texas, United States |
| Santa Gertrudis | 20 | Hybrid | Americas | Texas, United States |
| Luxi | 5 | Hybrid | Asia | Shandong Province, China |
| Qinchuan | 4 | Hybrid | Asia | Shaanxi Province, China |

Table S2. Descriptive statistics of the shared SNPs across bovine autosomes after marker harmonization and genome assembly conversion.

| Chr | Mean distance (kb) | Min distance (kb) | Max distance (kb) | n |
| --- | --- | --- | --- | --- |
| 1 | 272.63 | 13.56 | 1140.10 | 578 |
| 2 | 270.92 | 0.08 | 1546.48 | 501 |
| 3 | 280.55 | 1.19 | 1751.91 | 431 |
| 4 | 269.90 | 17.60 | 1305.77 | 443 |
| 5 | 276.60 | 20.04 | 1460.92 | 429 |
| 6 | 245.24 | 10.20 | 1196.90 | 479 |
| 7 | 286.24 | 2.25 | 2013.14 | 384 |
| 8 | 271.62 | 3.42 | 1477.42 | 409 |
| 9 | 282.77 | 15.57 | 1148.21 | 365 |
| 10 | 264.29 | 6.99 | 3110.26 | 387 |
| 11 | 281.97 | 4.79 | 1233.63 | 379 |
| 12 | 324.85 | 12.76 | 1937.47 | 263 |
| 13 | 282.56 | 0.38 | 2460.25 | 294 |
| 14 | 255.31 | 0.11 | 3739.42 | 322 |
| 15 | 301.60 | 20.85 | 1521.88 | 275 |
| 16 | 283.54 | 5.58 | 3115.80 | 276 |
| 17 | 277.88 | 9.80 | 1239.38 | 262 |
| 18 | 261.04 | 10.47 | 1232.28 | 250 |
| 19 | 247.10 | 1.37 | 1036.18 | 253 |
| 20 | 246.91 | 20.43 | 1164.53 | 290 |
| 21 | 275.10 | 20.14 | 1534.79 | 244 |
| 22 | 268.58 | 16.61 | 1226.69 | 222 |
| 23 | 253.88 | 20.72 | 1260.78 | 205 |
| 24 | 246.32 | 0.10 | 973.51 | 252 |
| 25 | 234.16 | 8.31 | 950.43 | 181 |
| 26 | 276.90 | 0.28 | 1223.48 | 186 |
| 27 | 258.71 | 20.03 | 1389.89 | 175 |
| 28 | 260.34 | 0.02 | 1305.82 | 173 |
| 29 | 271.11 | 3.08 | 1815.34 | 183 |

Table S3. K values tested in admixture analysis and their cross-validation error.

| K | Error |
| --- | --- |
| 12 | 0.453 |
| 9 | 0.453 |
| 14 | 0.453 |
| 13 | 0.453 |
| 11 | 0.453 |
| 7 | 0.454 |
| 10 | 0.455 |
| 8 | 0.455 |
| 15 | 0.456 |
| 6 | 0.457 |
| 16 | 0.460 |
| 5 | 0.462 |
| 17 | 0.464 |
| 4 | 0.465 |
| 18 | 0.465 |
| 19 | 0.468 |
| 20 | 0.469 |
| 3 | 0.470 |
| 21 | 0.471 |
| 2 | 0.476 |
| 23 | 0.477 |
| 22 | 0.477 |
| 25 | 0.488 |
| 26 | 0.491 |
| 24 | 0.492 |
| 27 | 0.496 |
| 28 | 0.503 |
| 29 | 0.509 |
| 30 | 0.520 |

Figure S1. Admixture cross validation error.
